# Therapeutical Treatment of Dental Pulps

**Published:** 1878-09

**Authors:** A. F. McLain


### THERAPEUTICAL TREATMENT OF DENTAL PULPS.

BY DR. A. F. M'LAIN, NEW ORLEANS.

*Read before the Southern Dental Association.*

Of the various pathological conditions which may arise in the teeth, none are more difficult to combat successfully, and none require a more intimate knowledge of the power of therapeutical agents, than those having their origin in the dental pulp. These organs are subject to irritation from various causes, growing out of certain circumstances, as accidents, thermometrical changes in temperature, and from that most common cause, decay of the teeth.

The effect of irritation to the pulp, if not met therapeutically at its earlier stage, ultimately leads to the congestion and inflammation of that organ, thereby producing its death; and by the continuance of these pathological states, disease is finally communicated to the peridental membrane. Now, the irritation, congestion, and inflammation of pulps, caused by mechanical shocks, fractures, abrasion of the dental fibers changes in temperature, or from the rapid progress of decay permitting chemical action on the pulp, are to be treated by different medicinal means suitable to the case. Congestion or inflammation having its origin from shocks, fractures, or abrasion, is not, according to the writer's experience, amenable to conservative treatment. In such cases no alternative remaining, the course to be pursued is to proceed to devitalize and extirpate the pulp, the operation to be concluded in the usual manner of root and crown filling. The chances, however, for preserving pulps alive in such cases are insignificant, and is successful only when the irritation is of a slight character, and when its origin has been due to gradual exposure, during the progress of which the vital forces, by throwing out plastic lymph, shield the pulp in a measure from the effects of external agents. When, on the contrary. the solution of the tooth substance is rapid, the pulp becom ing suddenly exposed, the amount of irritation which follows soon brings about that hyperæmic condition known as congestion, whereby more blood is thrown into the pulp cavity than is natural, and that surplus blood not finding an exit into the general circulation, produces so much pressure as to strangulate and cause the pulp to die. The consequences of this last do not stop here, for if not relieved at this stage, decomposition and liquefaction of the pulp tissues ensue, causing a development of gases, which, together with the decomposed matters, force themselves through the apical foramen, and there coming in contact with the pericemental or peridental membrane, set up anew the phenomena of congestive inflammation, and thus pave the way to the usual concomitants of that pathological condition, *alveolar abscess*, *necrosis*, etc. The sudden application of cold to the teeth acts in the same manner and with like results. This frequently occurs from the contact of ice cold water, the effect of which first drives the blood inward, in short inducing an intropulsive movement of the hæmal current, but which is soon followed by a reflex action, which fills to repletion the *neura-hæmal* sheath, thereby establishing a perfect stasis, with the usual phenomena and consequences of congestion.

A slight congestion of the pulp is sometimes removed by a gentle puncture, through a carious opening in order to evacuate the excess of blood; and should this fail, tepid water taken into the mouth, or a stimulating wash, may succeed in bringing about the desired end. Warmth, it is well known, acts beneficially in congestion by giving force to the arterial current, and in that way sweeps the stagnant venous fluid into the general circulation. Resort may be had also to local blood letting from the vicinity of the affected tooth, which acts very happily at times. A blister on the gum may be tried with propriety, but if immediate relief does not follow these several modes of medication, the vitality of the pulp is pretty sure to succumb, even though the hyperæmic condition had not lasted more than an hour or two. In speaking of local bleeding, preference should be given to leeching, as by that process the unloading of vessels in the immediate vicinity of those connected with the affected pulp is accomplished without leaving much irritation behind.

When an inflammatory process exists but in a moderate degree and of a small extent, that is merely the exposed portion of the pulp, the prognosis as regards the preservation of its vitality is more favorable, as the effusion into the pulp cavity is small in quantity, and the character of the exuded fluids being endowed with powers of organization, the vital forces are often able to limit the evil effects of inflammation; and even should this effort fail, the pulp organ may still be within therapeutical means. To gain so desirable an object, sedative applications should be made directly to the pulp and the surrounding parts, with the view of lessening the arterial supply, and to allow nature to resume her sway. For this purpose the strong preparation of aconite, combined with other agents, will be found in many instances quite efficacious. An irritably inflamed exposed pulp will frequently be relieved on applying a mixture consisting of equal parts of Fleming's tinct. of aconite and chloroform, with one-half their proportions of the aqueous solution (*Lugal's weaker preparation*) of iodine, to which may be added a few drops of creasote or carbolic acid. It is an error to flood a pulp with creasote or carbolic acid, as is commonly practiced. Although both of these substances possess anodyne properties, yet in an undiluted state they are escharotic and will prove irritants, and thus aggravate the symptoms. This application or dressing being made, the gums may be painted with the tinctures of aconite and iodine; a counter-irritant as well as a resolvent action is thereby obtained. Now, if the pulp is inclined to bleed, this should be encouraged by holding tepid water in the mouth or by syringing the cavity with it; and when the hemorrhage has ceased, the preceding anodyne dressing may be applied, and the cavity sealed with a solution of gum sandarac or mastic varnish on a pledget of cotton lint. Sometimes it happens that the pain continues, particularly if it proceeds from acidity; then an alkaline dressing, such as liquor potassa or soda, with the esssence of peppermint or tincture of camphor, may afford relief. A solution of chloral hydrate, made by triturating with it equal parts of gum camphor, to which may be added tincts. belladonna and aconite, or carvacrol, sometimes answers a good purpose in these cases. In fact, nearly all the essential oils will often relieve toothache arising from irritated or inflamed pulps. If by any of the measures indicated, the partial inflammation of a pulp has been subdued, something yet remains to be done in order to preserve the vitality of a pulp. And the benefit, nay, the absolute necessity which exists for the conservation of the entire vitality of a tooth as regards its proper nutrition and natural appearance, to say nothing of the gradual deterioration which inevitably befalls a crown when the *neuro-hœmal* connection of a tooth has been diseased, removed or destroyed, is too well understood by you, Mr. President and gentlemen, to need an elucidation here. Neither is it necessary to enter into a disquisition in regard to the peridental trouble, alveolar abscess, etc., which so often supervene upon the ulceration and putrefaction of a pulp when such a course has been allowed to take place.

To digress a little, many writers and speakers on this subject assert most emphatically that pulps in almost every stage of disease, from partial inflammation and ulceration up to actual gangrene, if limited, can be saved alive by a certain treatment. Now this is contrary to the experience of the author of this paper. For he has found that with the utmost care in the management, and in spite of the most thorough therapeutical treatment, a pulp that once has been the subject of inflammation, or in any way ulcerated, invariably dies, causing more or less subsequent disturbance.

As has already been intimated, the conservative treatment of a pulp is only half accomplished on arresting the irritation consequent upon exposure. It is essentially necessary to shield it from the contact of air, moisture and all extraneous exciting matters, else a recurrence of the same conditions would take place, and thus render subsequent efforts at preservation unavailing.

It is now pretty well admitted that pulps, if properly covered, and under favorable circumstances and in well contituted organs, especially in those cases where irritability is slight, or when the pulp has become exposed from the mechanical removal of caries, ninety-five percentum can be saved with all their vitality. To do this, some non-conducting material, and, at the same time, perfectly congenial, must be interposed between the exposed or sensitive pulp and the filling, and so adapted as to prevent pressure upon the pulp, as well as leave no space for the retention of air, or any exudation fluid that might escape from the pulp cavity. To these very causes may be attributed the common failures which formerly attended the *capping* of pulps when solid substances, such as gold caps, horn, lead, asbestos, etc., were employed for that purpose. A vacuum was thus left, which permitted plastic lymph, blood, or atmospheric air to accumulate or remain in contact with the pulp, and by that means caused continued irritation, which finally brought about its dissolution. Flooding the cavity with creasote or carbolic acid, and then coating the surface with oxychloride of zinc, often fails of its purpose, because the caustic effect of the chloride of zinc therein contained, is apt to irritate and cause the devitalization of the pulp, notwithstanding the thin film of coagulated albumen and fibrine that may have formed through the action of the creasote or carbolic acid upon the exuded plasma for which an affinity exists.

Now, this protecting layer may be more readily and effectually formed artificially by applying collodion to the exposed surface, but still more certainly by painting it over with a solution of Hill's stopping, which is obtained by dissolving the stopping in chloroform, forming a thin paste. In applying it, the cavity is to be well dried, and kept so; but previous to which, it should be bathed with the anodyne mixture previously mentioned; then, with a pointed stick or fine excavator armed with cotton lint, spread the solution of Hill's stopping over the exposed pulp or sensitive dentine, taking care to remove any surplus that might appear on the parietes of the cavity. It may be necessary, according to the size of the cavity and thinness of the solution, to coat over the surface several times in order to prevent the chloride from penetrating to the pulp. It will be found on trial that in ninety-nine cases of exposed pulps in the hundred treated in this way, not the slightest pain will be experienced on introducing the oxychloride of zinc. After this, sufficient time being given for the evaporation of the chloroform and for the film to attain some degree of firmness, a crust of oxychloride of zinc is spread over the surface to receive a permanent filling, or the cavity filled up entirely with the oxychloride as a temporary filling, as circumstances might require.

Perfect dryness of surface being an essential prerequisite for the successful application and cohesion of the solution of Hill's stopping, the rubber dam becomes an all-important aid in the process. Furthermore, the permanency of the film thus formed, as well as its non-conducting powers, are greatly enhanced by adding to the solution a small quantity of pulverized silex.

Not unfrequently, in cases of very slight pulp exposure, particularly if no previous irritation or inflammation had existed, the oxychloride covering may be dispensed with entirely, and a permanent filling inserted at once, after the simple covering of Hill's stopping has been made. On the other hand, where the double capping with the two sub stances is necessary in consequence of greater exposure, but otherwise under equally favorable conditions, very little risk will attend the conclusion of a permanent operation. In the event, however, of previous irritation having been manifested in pain or any other symptoms, prudence would naturally dictate the propriety of merely inserting a temporary filling after the cappings, with the view of waiting for future results, to see, in short, whether the vitality of the pulp has survived, or had in the meantime succumbed to morbid impressions.

Now, having treated at some length the mechanical means as well as detailed the remedial measures by which irritable and exposed pulps may be protected and medicated so as to preserve their vitality, it will not be inappropriate in the same connection to say something in reference to the therapeutical management of those pathological conditions which result from devitalized pulps.

They comprise *Alveolar Abscess*, *Pericementitis* or otherwise *Peridentitis*, *Exodontosis* and *Necrosis*. To appreciate fully the nature of such departures from health in the dental organs, it may be well for us to examine into their pathological causes, in order to be able to apply rational modes of treatment.

In the first place, any irritant, whether vital, chemical or mechanical, when applied to sentient parts, produces a disturbance in the circulation of that part. On its first application a rush of blood takes place—in another word, a determination—the artery dilates, becomes distended, but which subsides immediately if the irritant be withdrawn. On the other hand, the irritation being continued, the veins at and near this point soon manifest a tendency to become obstructed, the motion of the blood begins to flag, and if not removed, the circulation in the part ceases entirely. These being the usual phenomena presented in congestive hyperæmia as regards other organs, it can be readily apprehended that a precisely similar course follows in the vessels of a pulp; for in this state of hyperæmia, the artery being free to act from the *vis a tergo* force of the heart, continues to pump more blood through its channels into the pulp cavity, and the mechanical impediment offered to its flow into the venous opening fills to repletion the whole bony canal of the tooth, and thus produces so strong a pressure upon the vessels and pulp substance as to lead to the strangulation and ultimately to the death of this organ. Now, when that other and more complicated form of hyperæmia, inflammation, occurs, though located in the arteries and arising from the same cause, over-stimulation, still the same result eventually follows which is due to over distension, venous obstruction, stagnation, etc. Here then are two distinct processes, one *passive*, the other *active* hyperæmia, presenting different phenomena and with somewhat different results, when occurring in the soft textures, yet in the dental organs their pathological sequences are almost identical. The reason is obvious. In a healthy or physiological state, the artery and vein entering the tooth through a constringed apicial foramen, and all being enclosed in a common sheath with the nerve, they gradually expand at the bulbous portion of the pulp, and the blood of the artery, after having deposited its plastic nourishment in the different portions of the crown of a tooth, is returned freighted with the waste or worn-out materials into the vein in order to be conveyed back to the heart and lungs for subsequent aeration and revivification.

Either of these two processes, *passive* or *active* hyperæmia, occurring in other portions of the system endowed with expansive powers, the excess of blood finds a vent through the enlarged porous openings, made so by the overstretched fibrous net-work of which the channel-walls of the vessels are formed, thus affords them a partial relief, by the exudation of the serous portions of the blood into the surrounding tissues. Therefore, it may be readily perceived that an exposed pulp, being inclosed in a dense, unyielding, bony channel, an irritation occurring from any cause naturally induces an afflux of blood to the pulp, which so chokes or blocks up the avenues leading into the vein, as to fully establish all the phenomena of obstruction, stagnation, pressure, etc., characteristic of the congestive state, and which finally ends in the destruction of the *neuro*-hæmal structures.

Viewed pathologically, the distinctive differences existing between these two processes, *congestion* and inflammation, are sufficiently well marked as regards their respective symptoms and results when occurring in the soft tissues, as to be clearly appreciated. Preceded as they both are by determination, yet they have different locations, congestion being situated in the capillaries and veins, whilst the other is situated in the arteries. But one or the other may occur according to the strength of the irritant or the length of its duration. This has been demonstrated repeatedly under the microscope by applying to the web of a frog's foot a solution of capsicum; immediately blood was seen rushing toward the irritated point. Here was the determinative stage. On continuing the stimulation, though the arterial current had not ceased, yet its motion became somewhat retarded, the capillary tubes were observed to become more and more distended and of a darker red appearance, the blood corpuscles lost their disc-like form and were seen as elongated spheres worming themselves along, which imparted an oscillatory or vibratory motion to the local excess of sanguineous fluids. This was incipient inflammation. But, by increasing the strength of the irritant, or by prolonging its action still further, the veins were seen to be completely obstructed, the motion of the blood in the capillaries became arrested, and the part assumed a blue color. Here, then, were presented the usual phenomena attendant upon a fully established congestion. Now, what is the usual result of such hyperæmic effusions? The delicate membranes forming the channel-walls of those small hair vessels, from over-distension they naturally stretch, their fibers separate, and the serous fluids of the blood ooze through into the adjacent tissues. These fluids being strained as it were, of most of their organizable properties, and losing still further their vitality by their long retention in the parts, they naturally decompose, and in so doing, the textures thus encroached upon undergo atrophic softening, which favors the formation of abscesses.

The products of congestion being, as we have shown, *aplastic*, that is, devoid of the powers of organization by reason of their source, and from the degenerating process to which they had been subjected, would clearly account for the non-production of growths or hypertrophies as resulting from this state. And on the other hand, it is perfectly apparent why a moderate inflammation is more liable to become organized than the other; the effusion it furnishes, coming as it does immediately from the arterial system, is freighted with more plastic properties, hence has a tendency to organize. But an inflammatory turgescence may be so strong as to cause so great an afflux of blood to the part, that the exudation may be excessive, then the results may be the same as those yielded by congestion.

Viewed on general principles, the phenomenal characteristics and sequences of the several hyperæmias just considered, are ever the same in whatever portion of the system they may occur, excepting perhaps, in the dental organs, where they are somewhat modified and combined, owing no doubt to their greater density and want of elasticity and absorptive powers, thus giving these processes a compound character, which may be aptly termed *congestive-inflammation*. Consequently, whenever an irritation of sufficient magnitude occurs in a tooth, the hyperæmia excited commonly assumes a congestive type, and generally ends in the decomposition and liquefaction of the pulp tissues. The products thus yielded, pus and gas, find their way through the apical foramen, and there, coming in contact with the peridental membrane, renew the congestion or inflammation, which, if not subdued, eventually terminates in *alveolar abscesses* or in *necrosis*.

When the inflammatory congestion of a pulp is of a subacute nature, the foraminal infiltration is apt to be slight, hence the morbid process becomes limited to the end of the root, where a portion of peridental membrane detaches itself, and assumes a pouch-like form, which serves as a barrier to the extension of the irritation. Frequently the *vis conservatrix*, in thus limiting the morbid process by the formation of a receptacle for those acrid matters, gives vent to them by means of a fistulous opening in the vicinity of the root affected. Relief is thus afforded for a time, but sooner or later the peridental membrane loses its vitality, and then necrosis of the root or roots necessarily supervenes; and if these be not removed, the alveolar processes may become similarly involved.

It often happens that an inflammatory action in a pulp consequent upon its exposure, is of so mild a nature as to produce only a moderate degree of exudation, and being rich in plastic lymph, a deposition of secondary dentine takes place in the pulp-chamber, or on the roots, thus occasioning *exodontosis*.—*Transactions Am. Dental Association.*

TO BE CONTINUED.

